# Comparative Analysis of Bacterial Diversity and Composition in Oral Fluid from Pigs of Different Ages and Water Pipe Wall Biofilms

**DOI:** 10.3390/vetsci12111022

**Published:** 2025-10-22

**Authors:** Qinghai Ren, Wenlong Lu, Tingting Zhang, Shengkai Hao, Jiawen Wang, Xinrui Xu, Fei Wang, Zetong Huang, Xiaojing Lei, Shengliang Cao, Duanduan Chen, Yubao Li

**Affiliations:** 1 Phage Research Center, School of Agriculture and Biology, Liaocheng University, Liaocheng 252000, China; renqinghai113@126.com (Q.R.); luwenlong1@lcu.edu.cn (W.L.);; 2 School of Pharmacy and Food Engineering, Liaocheng University, Liaocheng 252000, China

**Keywords:** water supply network, metagenome, pigs at different stages, biofilm, microbial flora

## Abstract

**Simple Summary:**

This study explores the relationship between water pipe biofilms (slimy layers of microorganisms) in pig farms and the oral microbes of pigs at different ages. The key problem is that these biofilms can contaminate water and spread antibiotic-resistant bacteria; however, we lack an understanding of how pig age affects this process. The aim was to compare biofilms and oral fluids from 30-day, 70-day, and 110-day-old pigs using genetic sequencing. Results showed that the composition of biofilm bacteria changed with pig age—for example, *Brevibacterium* dominated in young pigs, while *Brevundimonas* was more common in older ones. Oral and biofilm microbes shared major bacterial groups like Actinobacteria, suggesting potential cross-contamination. A key antibiotic resistance gene (*adeF*) was found in all biofilms, especially in older pigs. These findings help us understand how microbes spread between pigs and their water systems, guiding strategies to reduce antibiotic resistance and improve water safety in pig farming.

**Abstract:**

Drinking water pipe biofilms, comprising viable microorganisms, microbial residues, and organic/inorganic particulates, pose significant risks to water safety by promoting the proliferation of opportunistic pathogens, pipe corrosion, and degradation of water quality. Their formation is strongly influenced by environmental conditions within the piping system. However, there is a lack of systematic research investigating the potential correlations between biofilm microbiota and the oral microbiomes of intensively farmed swine, as well as the age-dependent regulatory mechanisms shaping aquatic microbial communities. This pioneering study conducted a comparative analysis of biofilm microbiota from swine house water pipes and oral microbiomes across three growth stages (30-day BBF, 70-day NBF, and 110-day FBF groups), yielding three key findings. First, the biofilm biomass and dominant bacterial genera (e.g., *Brevibacterium* in BBF vs. *Brevundimonas* in FBF) exhibited stage-specific variations associated with swine age. Second, while the oral microbiomes showed no significant taxonomic divergence at the phylum or genus level, they shared characteristic phyla, including Actinobacteria and Bacteroidetes, with pipe biofilms, indicating potential cross-habitat microbial interactions. Third, the antibiotic resistance gene (ARG) *adeF* was consistently detected at high prevalence across all biofilm groups. These findings offer new insights into microbial transmission dynamics and inform risk mitigation strategies for livestock water supply systems.

## 1. Introduction

Biofilms in water distribution networks of pig farms are complex matrices composed of microorganisms, organic matter (including polysaccharides, proteins, nucleic acids, and lipids), and inorganic solids. These structures can protect bacteria from environmental stressors such as disinfectants and antimicrobial agents. Biofilm-associated bacteria typically account for over 95% of the total microbial biomass in water distribution systems across various facilities [1,2,3]. The composition of biofilm microbial communities is strongly influenced by environmental conditions, pipe materials, and water sources. Notably, oral microbes from livestock and poultry have been identified as key contributors to biofilm formation in farm water pipes. Previous studies have demonstrated that the gut microbiota of animals is modulated by various factors, including age [4], gender [5], environment [6], behaviors [7], and health care [7,8]. These factors can directly or indirectly affect microbial colonization in livestock water distribution systems. The damp, dark environment inside pipes provides an ideal niche for microbial adhesion and proliferation [9]. Microbial metabolism produces malodorous gases that may degrade farming conditions [6,10]. These formation dynamics are fundamentally influenced by hydraulic conduit parameters. However, significant knowledge gaps remain in understanding (1) the ecological connectivity between pipe-associated biofilms and porcine oral microbiota under intensive farming conditions and (2) the stage-specific mechanisms regulating aquatic microbial dynamics. Therefore, advancing our understanding of microbial succession within water distribution systems is essential for developing effective control strategies for pig farming.

In addition, water pipe biofilms serve as hotspots for antimicrobial resistance genes (ARGs). Prolonged antibiotic exposure in aquatic environments can induce the enrichment of ARGs, subsequently promoting the predominance and persistence of antibiotic-resistant bacteria (ARB) in the environment [11]. As emerging environmental contaminants, ARGs and ARB have attracted increasing attention in recent years [12]. Resistance genes may accumulate through the proliferation of ARB [13] and can also be amplified by horizontal gene transfer [13,14]. The enrichment of both ARGs and ARB poses an escalating threat to ecosystem integrity and public health [15]. Therefore, investigating the presence and distribution of resistance genes within biofilm-associated bacteria in livestock farm water systems is essential for assessing ecological and health risks [16].

This study employed metagenomic approaches to analyze the microbial composition within the water delivery systems of pig houses at different growth stages throughout the entire pig-rearing process. The primary objective was to elucidate the relationship between microbial communities residing on the inner surfaces of drinking water pipes and the age of the animals. In addition, the presence of ARGs and mobile genetic elements was examined. This study aimed to characterize the dynamic changes in microbial structure and functional potential within pipe-associated biofilms, with the findings providing a basis for developing targeted control strategies to reduce the prevalence of ARGs and ARB in livestock farming environments.

## 2. Materials and Methods

### 2.1. Sample Collection

The selected pig breed was the Duroc-Landrace-Yorkshire ternary commercial hybrid. Biofilm samples were collected from the water supply systems of three pig houses representing different growth stages: 30-day-old (BBF), 70-day-old (NBF), and 110-day-old (FBF). All pig houses used identical 304-grade stainless steel pipes, which were newly installed and subjected to thorough disinfection and cleaning prior to deployment to eliminate any pre-existing biofilm. Uniform tap water was introduced into all systems. After 90 d of feeding, biofilms were collected from the inner walls of the pipes. All pigs were fed An You brand feed via an automated feeding system, with free access to food and water. The environmental conditions were adjusted using a centralized control system to optimize the temperature and humidity according to pig age and behavior. During sampling, the inner pipe walls were rinsed with sterile PBS, and the biofilms were scraped using sterile cotton swabs. Sampling was performed at a height of 5–15 cm from the pig drinking nozzles with six sampling points per pig house. Each pig house was sampled in triplicate, resulting in a total of nine samples labeled as BBF1–3, NBF1–3, and FBF1–3. Microorganisms were extracted using 22 μm PVDF membrane filters, flash-frozen in liquid nitrogen, and stored at −80 °C. Each group consisted of three biological replicates. The collected samples were subsequently used for the metagenomic sequencing.

In each pig house of the same age group, three pigs were randomly selected for oral fluid collection using sterile cotton swabs. The samples were immediately flash-frozen in liquid nitrogen and stored at −80 °C. Nine oral fluid samples were collected from three age groups. Following sample processing, specimens were subjected to 16S rRNA gene amplicon sequencing. For Pipe biofilm (BBF/NBF/FBF), metagenomic sequencing is carried out on the Illumina platform (PE150) to analyze microbial composition, functional genes, and ARG distribution, with 9 target samples (3 groups × 3 repetitions). For Pig oral liquid (BBF/NBF/FBF), 16S rRNA gene amplicon sequencing is performed on the Ion S5TM XL platform to classify the composition and predict the function of oral microorganisms (via Tax4Fun), and there are 9 target samples (3 groups × 3 repetitions).

### 2.2. DNA Sample Detection

To sequence the variable regions of bacterial 16S rRNA genes on the MiSeq platform, the extraction of sufficient high-quality nucleic acids is essential. Genomic DNA was extracted from the sample using the CTAB method. Biofilm samples (membrane enrichment): The CTAB method was adopted, with a ratio of biomass to CTAB buffer of 1:10 (for example, 100 mg of biofilm precipitate added to 1 mL of CTAB buffer). The output volume of DNA after extraction was 50 μL. Oral fluid sample (cotton swab): The CTAB/SDS method was adopted. The ratio of the cotton swab eluent to the extraction buffer was 1:5 (200 μL of eluent added to 1 mL of extraction buffer), and the output volume was 30 μL. Quality control: The A260/280 values were detected by Nanodrop, with a qualified range of 1.8–2.0. Metagenomic library construction requires 1 μg of high-quality DNA (Qubit quantification), and 16S amplifiers require 1 ng/μL of DNA (verified by 1% agarose gel without degradation). Negative control: One blank control was set for each batch of extraction (only the extraction reagent was added, no sample), and it was confirmed after sequencing that there was no exogenous contamination. Positive control: Use genomic DNA of *E. coli* (DH5α) at a known concentration (10 ng/μL) as the extraction efficiency control to ensure the effectiveness of the extraction steps.

### 2.3. Construction and Sequencing of Library

Genomic DNA (1 μg) from each sample was used for library construction using the NEBNext^®^ Ultra DNA Library Prep Kit for Illumina (New England Biolabs, Ipswich, MA, USA). The prepared libraries were submitted to Beijing Novogene Technology Co., Ltd. (Beijing, China). for sequencing. DNA was fragmented to an average length of approximately 350 bp using a Covaris M220 ultrasonic disruptor (Covaris, Woburn, MA, USA). Library construction was performed according to the standard protocols. Preliminary quantification of the constructed libraries was conducted using a Qubit 2.0 Fluorometer (Thermo Fisher Scientific, Waltham, MA, USA), and the libraries were subsequently diluted to a concentration of 2 ng/μL. The insert size of each library was assessed using an Agilent 2100 Bioanalyzer (Agilent Technologies, Santa Clara, CA, USA). Upon meeting the expected size range, the quantitative PCR (qPCR) was performed to determine the accurate effective concentration of each library (with a threshold of >3 nM) to ensure quality. The libraries that passed quality control were pooled based on their effective concentration and target sequencing data volume, which were then subjected to PE150 on the Illumina platform.

### 2.4. Sequencing Data Quality Control, Assembly, and Analysis

Raw sequencing data should contain a proportion of low-quality reads. To ensure the accuracy and reliability of the downstream analyses, quality control and host sequence filtering were first performed to generate high-quality data, referred to as Clean Data. Metagenome assembly was then conducted using Clean Data from each sample. Gene prediction was performed on the assembled scaffolds using MetaGeneMark [2]. The predicted genes from all samples were subsequently merged to remove redundancy and construct a non-redundant gene catalog using CD-HIT (CD-HIT: accelerated for clustering the next-generation sequencing data). Based on this catalog, gene abundance profiles across samples were generated by mapping the Clean Data back to the unified gene set.

Starting from the gene catalog, each gene (Unigene) was annotated by comparison with the MicroNR database (version 2024-03) was used in conjunction with the BLASTP tool (version 2.17.0), with thresholds set as an e-value ≤ 1 × 10^−10^ and an identity ≥ 80%. This annotation was then integrated with gene abundance data to generate species abundance tables across various taxonomic levels. Functional analyses were conducted based on the gene catalog, including metabolic pathway annotation using the Kyoto Encyclopedia of Genes and Genomes (KEGG) database was annotated using the DIAMOND software (vO.9.9.110) with an e-value threshold of ≤1 × 10^−10^. For functional annotation based on orthologous groups with version v109.0. The eggNOG database (version v5.0) was employed with the eggNOG-mapper tool, using default settings, and functional characterization of carbohydrate-active enzymes using the Carbohydrate-Active Enzymes (CAZY). Based on the species and functional abundance tables, analyses, such as abundance clustering, PCA, NMDS, ANOSIM, and sample clustering, were performed. When the grouping information was available, Metastat and LEfSe multivariate statistical analyses were conducted, along with comparative pathway enrichment analysis, to identify the differences in species and functional composition between sample groups. Furthermore, ARGs were annotated using the Comprehensive Antibiotic Resistance Database (CARD), enabling the analysis of resistance gene abundance, associated microbial taxa, and resistance mechanisms.

### 2.5. Sequencing and Data Processing of Oral Microbial Genomic DNA

Genomic DNA from oral microbiota was extracted using the CTAB/SDS method. DNA purity and concentration were verified by 1% agarose gel electrophoresis, and the samples were diluted to 1 ng/μL with sterile water. 16S rRNA/18SrRNA/ITS genes of distinct regions (16SV4/16SV3/16SV3- V4/16SV4- V5,18SV4/18SV9, ITS1/ITS2, ArcV4) were amplified used specific primer (e.g., 16SV4: 515F-806R, 18SV4: 528F-706R, 18SV9: 1380F-1510R) with the barcode. The PCR amplification was performed in a 30 μL reaction system containing 15 μL of Phusion^®^ High-Fidelity PCR Master Mix (New England Biolabs), 0.2 μM primers, and 10 ng of template DNA. The thermal cycling conditions included an initial denaturation at 98 °C for 1 min, followed by 30 cycles of denaturation at 98 °C for 10 s, annealing at 50 °C for 30 s, and extension at 72 °C for 30 s, and a final extension at 72 °C for 5 min. Sequencing libraries were prepared using the Ion Plus Fragment Library Kit (Thermo Fisher Scientific, Waltham, MA, USA), quantified using a Qubit 2.0 Fluorometer (Thermo Scientific), and sequenced on the Ion S5^TM^ XL platform using single-end reads (400–600 bp). Bioinformatics processing included quality filtering using Cutadapt v1.9.1 and chimera removal by comparison with a reference database. Operational taxonomic units (OTUs) were clustered at 97% similarity using Usearch v7.0.1001, with dominant sequences selected as representatives. Taxonomic annotation was performed using the Mothur algorithm against the SILVA 132 SSU rRNA database, with a confidence threshold of 0.8 to 1.0, spanning from the phylum to species levels [17].

### 2.6. Oral Microbial Function Prediction Methods

Microbial functional profiling was predicted using Tax4Fun (version 0.3.1), which inferred the function based on 16S rRNA sequence homology using the nearest-neighbor method [18]. The prokaryotic 16S sequences from the KEGG database were aligned to the SILVA132, SSU Ref, and Nr databases using BLASTN (bit score >1500, version 2.17.0) to construct correlation matrices. The KEGG functional annotations generated by UProC or PAUDA were mapped to the SILVA132 database (http://www.arb-silva.de/ (accessed on 16 December 2024).) to derive the predicted microbial functional profiles.

### 2.7. Statistical Analysis

All statistical analyses were conducted using SPSS (version 31.0). The data were expressed as the mean ± standard error of the mean (SE), with a minimum of three biological replicates. Duncan’s multiple range test was employed as a post hoc procedure to assess statistical significance, with differences considered significant at *p* < 0.05. The enrichment differences between groups of functional pathways (such as KEGG) were analyzed by Metastat, and a *p*-value < 0.05 was considered significant enrichment.

## 3. Results

### 3.1. Pre-Feeding Waterline Bacteria Test

Sterility testing of the breeding waterline mesh was performed by collecting samples from multiple points within the waterline prior to the commencement of feeding. Bacterial cultures were subsequently conducted, and the results were confirmed at the beginning of the feeding experiment. These findings indicated that no bacterial contamination was detected within the internal structure of the waterline.

### 3.2. Quality Control of Data and Metagenome Assembly

Following the 90-day feeding period, the total microorganisms within the water pipes were collected, and high-throughput sequencing was performed on each sample to investigate microbial community dynamics. Quality control was applied to the raw sequencing data. The results showed that all samples generated more than 12,451.71 reads, with a maximum of 13,363.08 reads observed in sample NBF1. After the removal of low-quality reads, the range of clean data across all samples was between 12,428.16 and 13,341.93 reads. The GC content of all samples exceeded 54.04%. Furthermore, Q20 and Q30 scores were consistently above 97% and 92%, respectively. The metrics detailed in Table 1 demonstrated a high level of sequencing quality and offered a reliable basis for downstream analyses.

After preprocessing, clean data were obtained, and assembly analysis was conducted using MEGAHIT (v1.0.4) [19]. The results indicated that the BBF3 group exhibited the largest total scaffold length, reaching 772,220,995 bp, whereas the NBF3 group had the smallest total scaffold length of 287,773,422 bp. Similarly, the BBF3 group had the highest number of scaffolds, totaling 607,619, whereas only 21,356 scaffolds were assembled in the NBF3 group. The average scaffold length across all samples ranged from 1270 to 1489 bp. Detailed results are presented in Table 2.

### 3.3. Differences in Gene Distribution Among Samples from Three Pig Houses

**Figure 1:**
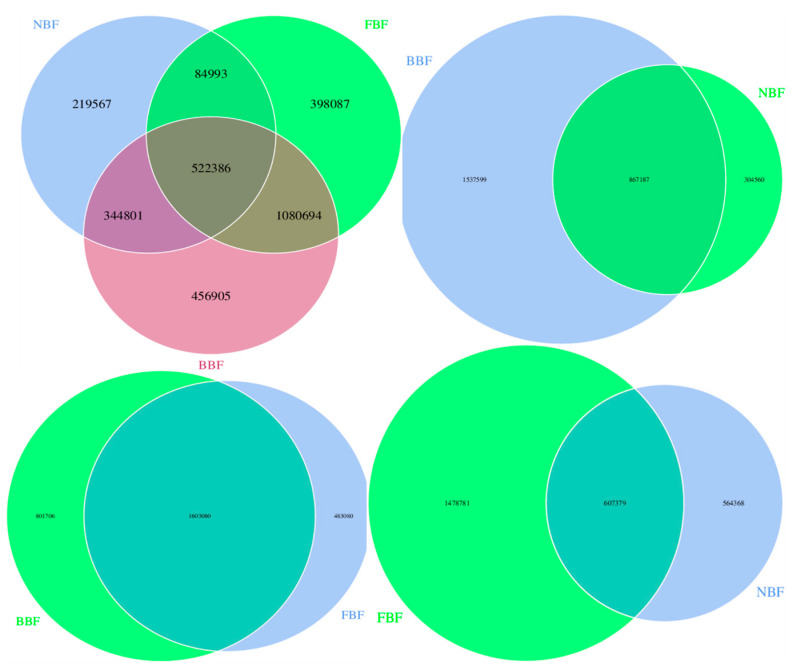
Venn diagram showing gene statistics among different groups.

In total, 84,993 differential genes and 522,386 shared genes were identified in the overlapping regions, with the BBF group exhibiting the highest number of unique genes (456,905). In contrast, the NBF group contained the fewest unique genes, with only 21,957 (Figure 1). These results indicated substantial genetic divergence between the BBF and NBF groups.

### 3.4. Correlation Analysis Between Samples Based on Gene Number

**Figure 2:**
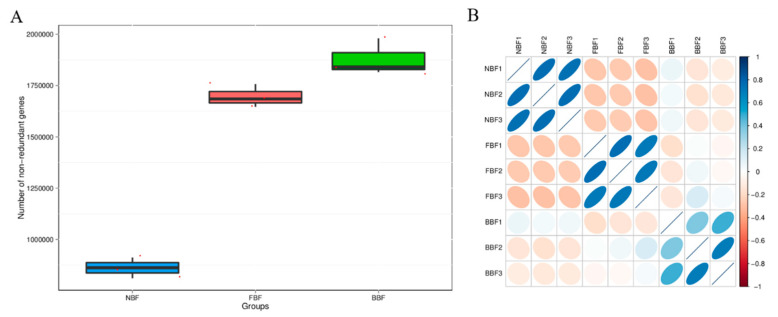
Sample correlation analysis results. (**A**) Box plot of differences in gene numbers between groups. (**B**) Heat map of correlation coefficient between samples.

In total, 219,567 non-redundant genes were identified in the NBF group, 398,087 in the FBF group, and 456,905 in the BBF group (Figure 2A and Table 3). The gene abundance correlation among samples served as a key metric to evaluate experimental reliability and appropriateness of sample selection. A correlation coefficient approaching 1 indicated a high similarity in gene abundance patterns across samples. Spearman correlation analysis was conducted to assess reproducibility and inter-sample relationships. The results revealed that inter-group variation was markedly greater than intra-group variation. Notably, the biofilm microbial communities on water pipe walls demonstrated significant differentiation across the three pig houses, with much smaller differences observed within individual groups. A particularly strong correlation was detected between samples FBF3 and BBF2. These observations validated the grouping strategy and underscored the reliability and analytical value of the dataset for subsequent investigation.

### 3.5. Taxonomic Analysis

**Figure 3:**
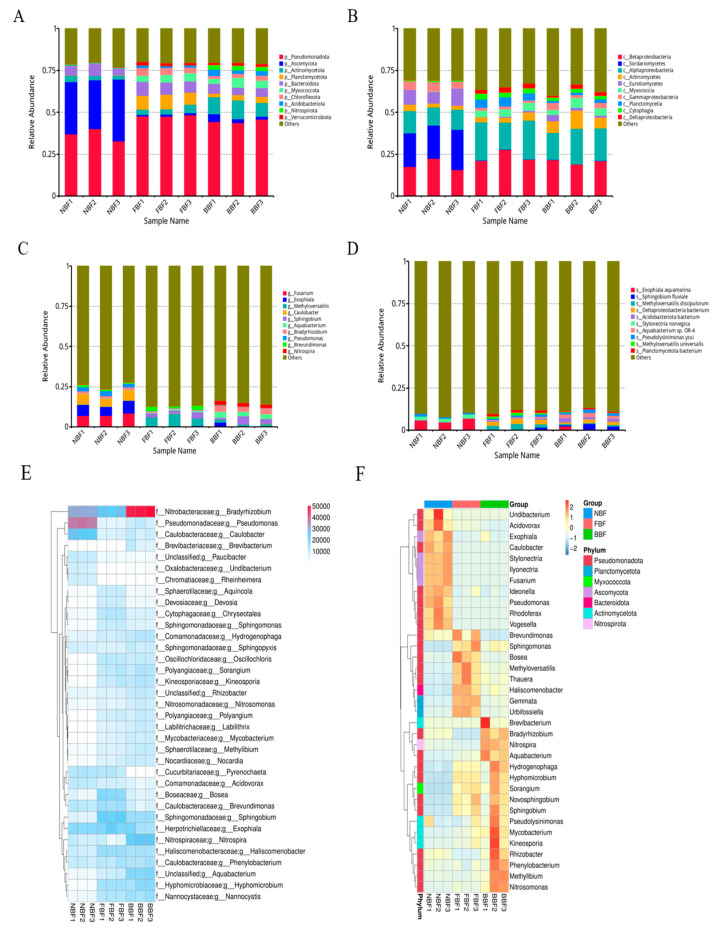
Phylogenetic composition of microbial communities. (**A**) Phylum-level distribution; (**B**) class-level distribution; (**C**) genus-level distribution; (**D**) species-level distribution; (**E**) heat maps and hierarchical clustering of 35 dominant genera; (**F**) cluster analysis of dominant genera in the three groups of samples.

Based on the filtered taxonomic data, the top 10 species with the highest relative abundance in each group were identified and visualized in Figure 3, with all remaining taxa grouped under the category “Others”.

Based on Krona analysis [20], *Pseudomonadota*, *Ascomycota*, and *Actinomycetota* were identified as the dominant phyla across all groups, with a significant reduction in richness observed in the NBF group compared with the other two groups (*p* < 0.05) (Figure 3A). At the class level, *Betaproteobacteria*, *Sordariomycetes*, and *Alphaproteobacteria* were predominant in each group, reflecting patterns consistent with the corresponding phyla. Among these, *Pseudomonadota* was the most abundant class in all three groups (Figure 3B). Similarly to the phylum level, class-level richness in the NBF group was significantly lower than that in the other two groups (*p* < 0.05). To further characterize microbial composition, relative abundance tables at multiple taxonomic levels were generated. The 35 most abundant genera were selected, and their abundance values across all samples were visualized in a heatmap with hierarchical clustering conducted at the species level to enhance interpretability. As shown in Figure 3E, the BBF group was dominated by *Nitrobacteraceae* and *Bradyrhizobium*, while *Pseudomonadaceae* and *Pseudomonas* were predominant in the NBF group. In contrast, the FBF group exhibited relatively uniform gene distribution among species. Cluster analysis of the dominant genera in the three groups (Figure 3F) revealed that the NBF group primarily harbored *Acidovorax*, *Exophiala*, *Caulobacter*, *Stylonectria*, *Ilyonectria*, *Fusariumideonella*, *Pseudomonas*, *Rhodoferax* and *Vogesella*. The FBF group was characterized by the presence of *Brevundimonas*, *Sphingomonas*, *Bosea*, *Methyloversatilis*, *Thauera*, *Haliscomenobacter*, *Gemmata*, and *Urbifossiella*. In the BBF group, dominant taxa included *Brevibacterium*, *Bradyrhizobium*, *Nitrospira*, *Aquabacterium*, *Hydrogenophaga*, *Hyphomicrobium*, *Sorangium*, *Novosphingobium*, *Sphingobium*, *Pseudolysinimonas*, *Mycobacterium*, *Kineosporia*, *Rhizobacter*, *Phenylobacterium*, *Methylibium*, and *Nitrosomonas*.

At the genus level, *Fusarium* was significantly more abundant in the NBF group compared to the BBF and FBF groups (*p* < 0.05). At the species level, the relative abundance of *Sphingobium fluviale* was markedly elevated in the BBF group (*p* < 0.05), whereas *Exophiala aquamarina* was significantly enriched in the NBF group (*p* < 0.05). In contrast, Deltaproteobacteria represented the dominant species in the FBF group, with a significantly higher abundance than in the other groups (*p* < 0.05) (Figure 3D).

Sphingomonas and *Fusarium* were identified as the dominant genera in the biofilms sampled from the water pipes in the BBF and NBF groups, respectively. Leão et al. [21] reported that Sphingomonas functioned as an early colonizer in water pipe systems and maintained dominance throughout biofilm development. The isolates of *Sphingomonas* from membrane environments exhibited distinct adaptive characteristics, including enhanced motility and elevated tolerance to various environmental stressors such as temperature fluctuations, ionic strength, and pH variability.

### 3.6. Phylogenetic Analysis

**Figure 4:**
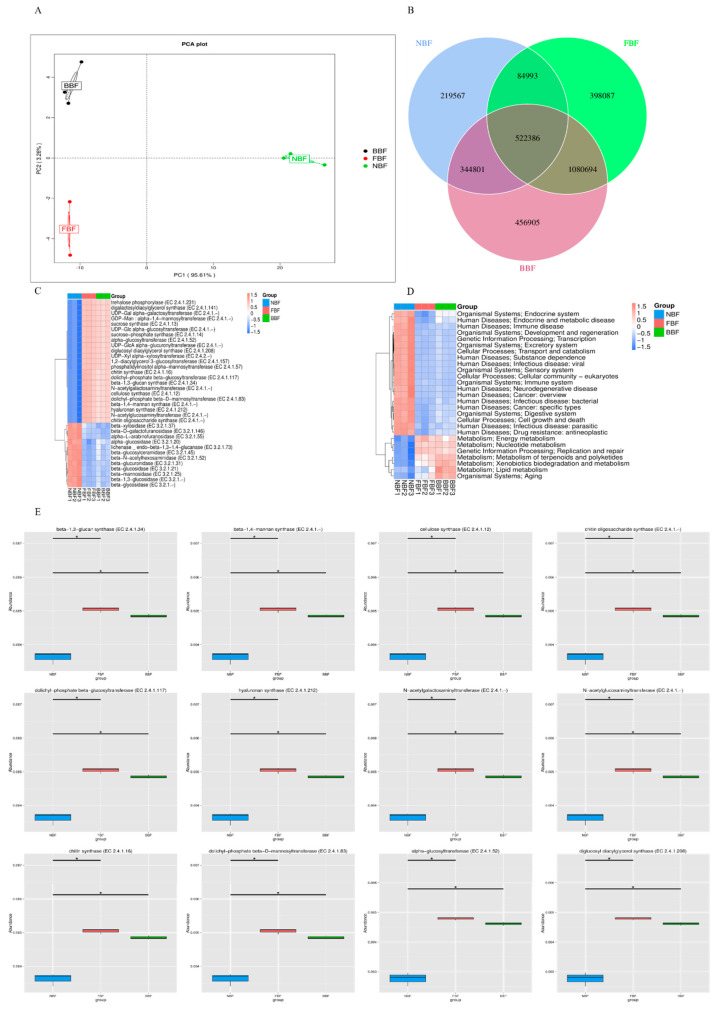
Analysis of potential functional attributes of microbial communities. (**A**) PCoA results of all samples; (**B**) heat map of KEGG pathway analysis; (**C**) CAZy annotation results; (**D**) box plot of differential metabolic pathway enrichment; (**E**) Significant difference function box diagram display. “*” indicates a significant difference between the two groups (q value < 0.05).

To investigate the potential influence of animal age on microbial communities within water pipe biofilms, the functional annotation was performed using the KEGG and the CAZY database [22]. Principal coordinate analysis (PCoA) revealed significant compositional differences among the groups (*p* < 0.05) (Figure 4A). KEGG pathway analysis indicated a significant enrichment (*p* < 0.05) in lipid metabolism and aging-related functions. Compared to the NBF group, the pathways associated with endocrine and metabolic disorders, immune diseases, development and regeneration, transcription, transport and catabolism, viral infectious diseases, eukaryotic cellular communities, and cell growth were more prominently enriched in both the BBF and FBF groups (Figure 4B). The CAZY analysis further identified the significant alterations in metabolic enzymes, including alpha-glucosyltransferase, beta-1,3-glucan synthase, beta-1,4-mannan synthase, cellulose synthase, chitin oligosaccharide synthase, chitin synthase, diglucosyl diacylglycerol synthase, dolichyl-phosphate beta-D-mannosyltransferase, and dolichyl-phosphate beta-glucosyltransferase across the microbial communities of the BBF, NBF, and FBF groups.

### 3.7. ARGs Analysis

**Figure 5:**
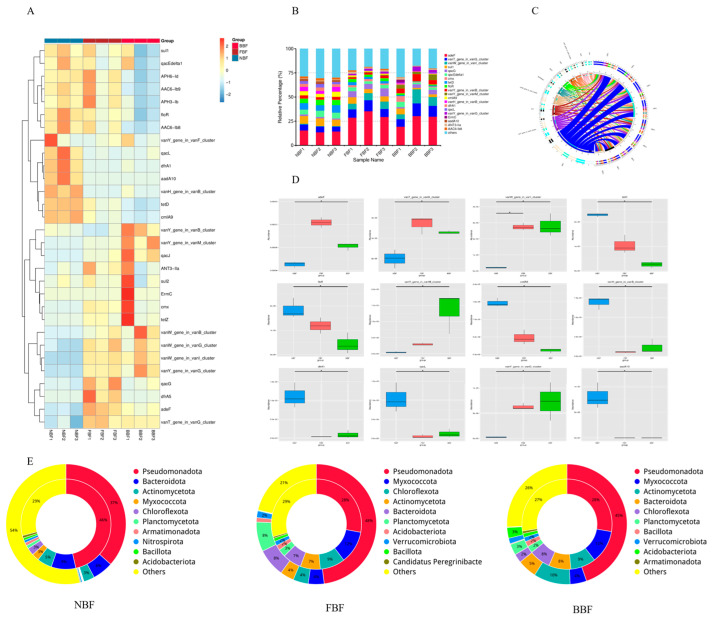
Prediction results of microbial antibiotic resistance genes. (**A**) Heatmap of the top 20 ARO abundances in each sample; (**B**) Bar chart showing the percentage of the top 20 AROs in each sample; (**C**) Circular overview of resistance genes across samples; (**D**) Box plot showing the proportion of resistance genes in different groups; (**E**) Distribution of resistance genes by species. The inner circle represents the species distribution of AROs, and the outer circle represents the species distribution of all genes in the group. “*” indicates a significant difference between the two groups (q value < 0.05).

The Antibiotic Resistance Ontology (ARO) framework was employed for ARGs by using the CARD. A total of 30 ARGs were identified (Figure 5A). Among them, the top 20 included *adeF*, *vanT* (*vanG* cluster), *vanW* (*vanI* cluster), *sul1*, *qacG*, *qacEdelta1*, *cmx*, *tetD*, *floR*, *vanY* (*vanB* and *vanM* clusters), *cmIA9*, *vanH* (*vanB* cluster), *dfrA1*, *qacL*, and *vanY* (unspecified van cluster). The relative abundances of ARGs across all samples were quantified (Figure 5C,D), with *adeF* consistently exhibiting the highest abundance, particularly in the FBF group. Although the resistance gene *cmx* was detected, its abundance remained minimal. Notably, the FBF group exhibited the highest overall ARG abundance, indicating potential age-dependent enrichment of ARGs. To investigate the origins of ARGs, bacterial species contributing to ARGs were annotated (Figure 5E). In the NBF group, ARGs were predominantly associated with *Pseudomonadota* (37%), *Bacteroidota* (6%), and *Actinomycetota* (3%). In contrast, the FBF group displayed greater taxonomic diversity, with ARGs attributed to *Pseudomonadota* (48%), *Myxococcota* (4%), *Chloroflexota* (4%), *Actinomycetota* (4%), *Bacteroidota* (8%), and *Verrucomicrobiota* (2%). The BBF group exhibited a diversity profile comparable to that of the FBF group but showed a relatively greater contribution from Actinomycetota. These findings suggest that the composition and abundance of ARGs in water pipe biofilms are largely shaped by the local microbial community.

### 3.8. Amplicon Detection of Saliva Samples from Pigs at Different Stages

**Figure 6:**
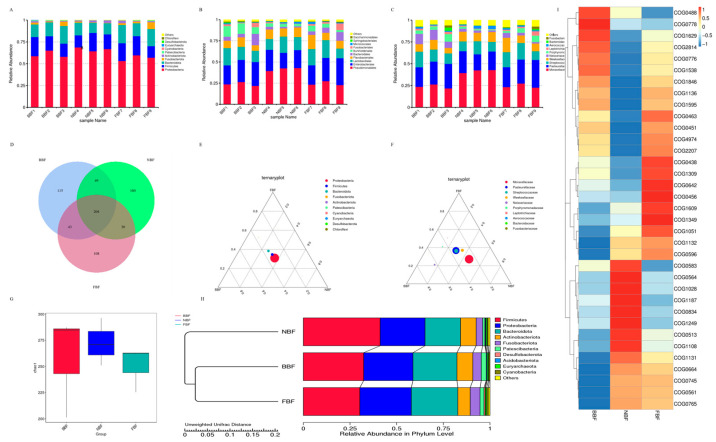
Amplicon-based detection of saliva samples from pigs at different growth stages. (**A**) Species richness at the phylum level; (**B**) species richness at the order level; (**C**) species richness at the family level; (**D**) Venn diagram of saliva samples from pigs at three stages; (**E**) ternary plot of phylum-level composition; (**F**) ternary plot of family-level composition; (**G**) analysis of differences in alpha diversity indices; (**H**) UPGMA (Unweighted Pair Group Method with Arithmetic Mean) clustering tree; (**I**) clustering analysis of relative abundances based on PICRUSt2 functional annotation.

Amplicon sequencing technology was used to perform a comprehensive analysis of the oral microbiota in pigs across distinct developmental stages, with the primary objective of elucidating the taxonomic composition and community characteristics of these microbial ecosystems. At the phylum level, *Proteobacteria*, *Firmicutes*, *Bacteroidota*, *Fusobacteriota*, and *Actinobacteriota* were consistently identified as dominant taxa across all three stages (Figure 6A). At the order level, the predominant microbial groups included *Pseudomonadales*, *Enterobacterales*, *Lactobacillales*, *Bacteroidales*, *Fusobacteriales*, and *Actinomycetales* (Figure 6B). The analysis at the family level revealed *Moraxellaceae*, *Pasteurellaceae*, *Streptococcaceae*, *Weeksellaceae*, *Neisseriaceae*, and *Porphyromonadaceae* as the major contributors to the microbial community structure (Figure 6C). Functional profiling of oral microbiota was conducted using the PICRUSt2 software package. Based on 16S rRNA sequencing data, functional annotation and abundance information were derived through alignment with multiple databases, including KEGG, COG, PFAM, TIGRFAM, and MetaCyc. The 35 most abundant functional categories were selected for visualization via heatmap generation and clustering analysis, thereby elucidating functional differentiation among samples.

Overlapping analysis of saliva samples revealed that 204 OTUs were shared among the three age groups. The NBF group exhibited the highest number of unique OTUs (160), followed by the BBF (115) and FBF (108) groups (Figure 6D). Based on these findings, ternary phase diagrams were constructed at the phylum and family levels. At the phylum level, Proteobacteria accounted for the largest proportion, followed by *Firmicutes* and *Bacteroidota* (Figure 6E). At the family level, *Moraxellaceae* exhibited a relatively high abundance in both FBF and BBF groups (Figure 6F). For species clustering analysis, the unweighted pair group method with arithmetic mean (UPGMA) revealed clear intra-group consistency, with the NBF group showing the lowest within-group variation (Figure 6G,H). Based on these clustering results, the functional potential of microbial communities in different groups was predicted. As shown in Figure 6I and Table 4, the BBF group was predominantly enriched in functional categories, such as COG0488, COG0778, COG1629, and COG2814. In the NBF group, the dominant functions included COG0583, COG0564, COG1028, COG1187, COG0834, and COG1249. The FBF group exhibited functional advantages for COG0438, COG1309, COG0642, COG1609, COG1349, and COG1051. These predicted microbial functions are primarily involved in physiological processes including substrate transport, antibiotic resistance, metabolic regulation, and immune modulation.

## 4. Discussion

This study investigated the diversity of microbial communities in the inner walls of water pipes in pig houses at different growth stages. A series of experimental analyses was conducted, including assessments of microbial distribution across three pig house groups, correlation analysis of gene abundance among samples, systematic taxonomic evaluation, and profiling of ARGs. Metagenomic analysis of biofilms on water pipe surfaces provided a comprehensive overview of the total genomic content of microbial populations, offering deeper insights into community composition, microbial interactions, and metabolic pathways. In comparison, amplicon sequencing of porcine oral microbiota targeted specific gene regions to identify the microbial taxa and quantify their relative abundances.

Owing to variations in surface roughness and physicochemical characteristics, biofilms formed on different pipe materials in water distribution systems exhibited considerable compositional differences. Pipe material properties have been recognized as critical determinants of biofilm development [23]. In this study, all water pipes were composed of 304-grade stainless steel, and the dominant bacterial genera identified across the three age groups were *Fusarium* and *Exophiala*, which differed markedly from those typically observed in PVC and STS samples. In contrast, PVC and STS pipes have been shown to support a higher abundance of stalked bacterial genera such as Hyphomicrobium, which difference may be due to the fact that the surface smoothness of 304 stainless steel (Ra = 0.2 μm) is lower than that of PVC (Ra = 0.05 μm), which is more conducive to the colonization of fungi (such as *Fusarium*) [24].

Functional prediction analysis revealed significant alterations in the activities of glycoside hydrolases and auxiliary enzymes in the NBF group. A previous study demonstrated that the expression levels of key enzymes involved in carbohydrate and amino acid metabolism in piglet intestinal microbiota were markedly regulated in response to bacterial infection [5]. In contrast, glycosyltransferases and carbohydrate esterases were predicted to be predominantly altered in the BBF and FBF groups. Glycoside hydrolases are known to play essential roles in the synthesis of oligosaccharides, alkyl glycosides, and aromatic glycosides, and also function as a microbial defense mechanism by the glycosylation of certain antibiotics [25]. Some pathogens produce glycoside hydrolases that degrade host-derived sugars during the process of nutritional acquisition. Although various glycosyltransferases have been associated with the modulation of antibiotic biosynthesis, the specific biological functions of the enzymes identified in this study require further investigation. Collectively, these findings suggest that stage-specific microbial communities contribute to distinct metabolic profiles.

Multiple factors may influence the formation of microbial communities during pig-rearing. In this study, a distinct microbial shift was observed in the NBF group, which is consistent with the findings of Bishnu Adhikari [26] who reported a significant increase in microbial diversity following weaning. Prior studies have confirmed that antibiotic exposure can alter the intestinal microbiota composition [27]. For instance, ref. [28] demonstrated dynamic changes in bacterial communities in pigs, aligning with our observations that the oral microbiota could affect the surrounding environment, including water delivery systems, during drinking. Other investigations have similarly indicated stage-specific differences in intestinal microbiota, which can directly induce structural shifts in microbial communities within both pigs and their environments. Therefore, the pig house environment and oral microbiota may contribute to variations in the microbial richness of water pipe biofilms, warranting further research.

In the analysis of ARGs, *adeF* was identified as the most abundant ARG across all three groups. Lee K. Kimbell et al. reported that *blaTEM* was the predominant ARG in biofilms formed on iron water pipes [29], suggesting that ARG profiles may be influenced by environmental factors, including the pipe material and the frequency of antibiotic exposure in water systems. The high prevalence of *adeF*, particularly in the FBF (110-day) group, is consistent with its role as a major efflux pump gene. The *adeF* gene has been reported to be associated with resistance to β-lactam and fluoroquinolone antibiotics [30]. In this study, this gene was detected in high abundance in all biofilm samples, especially in the FBF group, suggesting that it may be involved in the antibiotic resistance phenotype in this environment. However, the specific resistance phenotype needs to be further verified through bacterial isolation and culture, as well as drug sensitivity tests. This pattern may reflect prolonged antibiotic exposure during the finishing phase, when β-lactam antibiotics are commonly administered via feed or drinking water to prevent post-weaning infections. The FBF group exhibited the highest total ARG abundance (Figure 5D), likely due to the cumulative antibiotic pressure at later growth stages. Notably, *adeF*-harboring bacteria, such as *Pseudomonas* and *Brevundimonas*, were dominant in FBF biofilms (Figure 3F), suggesting that this resistance mechanism may confer a selective advantage under sustained antibiotic stress. These findings underscore the necessity for targeted surveillance of *adeF*-mediated resistance in swine water systems, particularly during the fattening phase when antibiotic application is typically intensified [7]. However, this study did not directly detect the concentration of antibiotics in feed or drinking water. This speculation needs to be further verified in combination with high-performance liquid chromatography detection in the future.

Comparative analyses revealed the presence of shared bacterial taxa between porcine oral microbiota and water pipe biofilm communities. Notably, *Escherichia coli* was concurrently identified through amplicon sequencing of oral samples and metagenomic analysis of pipe-associated biofilms. As a typical commensal of the intestinal tract, *E. coli* may be introduced into the oral cavity via the ingestion of contaminated water, if present on biofilm-coated pipe surfaces [31,32]. In this study, oral microorganisms and water pipe biofilms shared part of the microbiota, suggesting that there might be microbial exchange between the two. However, at present, only the co-occurrence of the microbiota can be confirmed, and the transmission direction cannot be determined. The exchange mechanism will be further verified through fluorescence labeling and tracing or dynamic sampling experiments in the future. Hygiene conditions within the pig housing environment, combined with feeding and drinking behaviors, can provide favorable conditions for the persistence of this microorganism in both oral and biofilm-associated microhabitats.

In addition, certain Staphylococcus species, particularly Staphylococcus aureus, were detected in both oral and water pipe biofilm microbial communities. Staphylococcus aureus is characterized by a high level of environmental adaptability, allowing its survival across a wide range of ecological niches [33]. The warm and humid conditions prevalent in pig housing environments offer a conducive habitat for its proliferation [34]. Furthermore, the routine behavioral patterns of pigs may facilitate bidirectional microbial transmission between the oral cavity and pipe-associated biofilms.

Despite the presence of several shared species, notable differences were observed between the oral and water pipe membrane microbial communities. *Streptococcus salivarius* is a predominant member of the oral microbiota and is known for its strong association with the oral mucosal surface [35]. This species has evolved to thrive under specific physiological conditions in the oral cavity, including elevated salivary amylase activity, stable temperature, and high humidity, by utilizing salivary nutrients to support its growth and colonization [36]. In contrast, it has been identified to be relatively underrepresented in the biofilm microbiota of water pipes, likely due to the starkly different physicochemical environment, which is less favorable for its survival and proliferation.

Conversely, bacteria exhibiting strong biofilm-forming capacities, such as *Pseudomonas aeruginosa*, were discovered to be more prevalent within the water pipe membrane microbiota. The ability of *Pseudomonas aeruginosa* to secrete extracellular polysaccharides facilitates the establishment of a stable biofilm matrix on the inner surfaces of water pipes. This matrix serves dual functions: providing mechanical protection against hydraulic shear forces and enhancing nutrient acquisition. However, within the porcine oral cavity, the dynamic nature of the microenvironment, characterized by salivary flow, host immune defenses, and microbial competition, imposes constraints on the colonization and proliferation of P. aeruginosa.

Functional annotation analysis, particularly based on the KEGG metabolic pathway database [37], revealed notable functional similarities between the microbial communities of the pig oral cavity and those of the water pipe membranes. Both communities harbored genes associated with carbohydrate and energy metabolism, indicating that fundamental metabolic processes were essential for microbial survival irrespective of the environmental niche. Notably, genes involved in the glycolytic pathway were identified in both habitats, facilitating carbohydrate utilization for energy production. This functional convergence suggests that microorganisms occupying distinct ecological niches may undergo evolutionary selection for similar functional gene repertoires to meet comparable metabolic demands.

Nevertheless, notable functional differences were identified between the two microbial communities. Microorganisms inhabiting the pig’s oral cavity exhibited greater involvement in processes related to host immune regulation and the initial stages of food digestion [38], which is consistent with the oral cavity’s role as the primary site of food intake and enzymatic breakdown. For instance, Lactobacillus species isolated from the oral microbiota were found to secrete organic acids, particularly lactic acid, which modulates the pH of the oral microenvironment. This acidification not only suppressed the proliferation of pathogenic bacteria but also facilitated the preliminary digestion of dietary substrates [39].

In contrast, microorganisms inhabiting water pipe membranes possess distinct functional genes associated with nutrient assimilation from the aqueous environment, resistance to hydraulic shear stress, and surface adhesion [40]. Notably, genes encoding extracellular polysaccharide synthases were frequently detected, which are critical for the synthesis and structural maintenance of biofilms on pipe surfaces. These genes facilitate the development of robust biofilm architecture, thereby ensuring the persistence and ecological stability of microbial communities within the pipe infrastructure.

Longitudinal investigations of pig populations at various developmental stages have demonstrated a significant correlation between oral microbiota and microbial communities colonizing water pipe membranes within pig housing facilities. As pigs advance through successive growth phases, dynamic shifts occur in the composition and structure of their oral microbiota [41]. Simultaneously, corresponding alterations were observed in the microbial communities adhering to water pipe membranes. During the early developmental stages, both oral and pipe-associated microbiota exhibit a relatively low complexity. With the progression of growth, an increase in microbial diversity was consistently observed in both habitats.

This synchronized shift in microbial diversity can be attributed to dynamic physiological and behavioral changes associated with pig growth. As pigs mature, alterations in feeding behavior, water consumption patterns, and environmental interactions lead to modifications in the composition of the oral microbiota. Concurrently, the pigsty environment, including microbial communities inhabiting water pipe membranes, undergoes stage-specific changes influenced by animal development and varying management practices. These processes are mutually interactive, reinforcing the observed co-variation between the two microbial niches [42].

In summary, the oral microbiota of pigs and the microbial communities residing on water pipe membranes within pigsties demonstrated both shared and distinct characteristics. The presence of common microbial taxa and functional profiles can largely be attributed to pig behavior, such as feeding and drinking, as well as environmental factors within the pigsty. However, the distinct ecological conditions of the oral cavity and water pipe surfaces contribute to pronounced differences in the microbial composition and metabolic functions [43]. The dynamic and reciprocal relationship between these two microbiomes, modulated by developmental stages and environmental changes, highlights their integrated role in shaping the broader microbial ecology of the pig-rearing environment.

Future studies should aim to elucidate the underlying mechanisms governing these inter-microbial interactions. A deeper understanding of these processes is critical for optimizing environmental management strategies and improving swine health. For example, targeted interventions to modulate biofilm microbial communities in water delivery systems may provide a novel approach to suppress the proliferation of pathogenic organisms, thereby reducing the risk of disease transmission and supporting healthy pig development.

In conclusion, this study employed microbiome sequencing to investigate the microbial communities attached to water pipe membranes in three pig houses at distinct growth stages (BBF, NBF, and FBF). Metagenomic analyses were conducted to characterize bacterial distribution, predict microbial functions, and identify ARGs. These findings could offer a valuable foundation for exploring how pig age and environmental conditions influence biofilm formation in livestock water systems. This study revealed the influence of pig age on the microbial composition and ARG abundance of water pipe biofilms. It was found that the shared microbiota between the oral cavity and the biofilm suggested the potential for microbial exchange, and the *adeF* gene was highly abundant during the fattening stage. These results provide a basis for analyzing the microbial risks in the water supply system of pig farms, but it should be noted that: (1) The direction of microbial transmission has not yet been clearly defined; (2) Other sources of ARG, such as feed, water sources and environmental diffusion, apart from the oral microorganisms of pigs, need further study; (3) The influence of other environmental factors (such as antibiotic residues) was not taken into account. Subsequent research is needed to address the above issues and formulate more precise control strategies for biofilms and ARGs.

## Figures and Tables

**Table 1:** Summary of sample data quality control statistics.

Sample	Insert Size (bp)	Raw Data	Clean Data	Clean Q20	Clean Q30	Clean GC (%)
NBF1	350	13,363.08	13,341.93	97.58	93.47	54.56
NBF2	350	12,937.60	12,916.54	97.52	93.35	54.04
NBF3	350	12,710.49	12,688.42	97.57	93.51	53.66
FBF1	350	13,019.04	13,000.19	97.34	93.07	61.09
FBF2	350	12,451.71	12,428.16	97.31	92.94	61.41
FBF3	350	12,998.80	12,970.01	97.52	93.48	61.84
BBF1	350	12,957.69	12,926.97	97.61	93.38	60.17
BBF2	350	12,974.73	12,939.75	97.66	93.55	62.41
BBF3	350	13,330.60	13,292.03	97.68	93.64	61.51

**Table 2:** Summary of basic scaffold assembly statistics for each sample.

Sample ID	Total Length (bp)	Number of Scaffolds	Average Length (bp)	N50 Length (bp)	N90 Length (bp)	Max Length (bp)
NBF1	348,804,097	250,672	1391.48	1791	601	259,671
NBF2	359,763,692	260,253	1382.36	1752	602	130,849
NBF3	287,773,422	217,356	1323.97	1587	596	200,836
FBF1	677,294,730	459,987	1472.42	2037	599	639,520
FBF2	626,882,810	421,083	1488.74	2061	603	694,772
FBF3	684,005,321	494,497	1383.23	1696	596	397,351
BBF1	662,760,463	520,064	1274.38	1426	577	578,482
BBF2	740,577,707	533,990	1386.88	1744	606	298,203
BBF3	772,220,995	607,619	1270.90	1466	589	389,864

**Table 3:** Summary of gene statistics for each sample.

Sample	NBF1	NBF2	NBF3	FBF1	FBF2	FBF3	BBF1	BBF2	BBF3
Gene_number	912,055	862,381	811,137	1,684,947	1,646,640	1,757,003	1,816,062	1,840,404	1,980,463

**Table 4:** Statistical analysis of differential enrichment of microbial functions.

COG ID	Description
COG0488	ABC cassette proteins with duplicated ATPase domains, Uup/ABCF family
COG0778	Nitroreductase
COG1629	Outer membrane receptor protein, Fe transport
COG2814	Predicted arabinose efflux permease AraJ, MFS family
COG0776	DNA-binding chromatin protein HU or IHF, alpha or beta variants
COG1538	Outer membrane protein TolC
COG1846	DNA-binding transcriptional regulator, MarR family
COG1136	ABC-type lipoprotein targeting system ATPase component LolD
COG1595	DNA-directed RNA polymerase specialized sigma subunit, sigma24 family
COG0463	Glycosyltransferase involved in cell wall bisynthesis
COG0451	Nucleoside-diphosphate-sugar epimerase
COG4974	Site-specific tyrosine recombinase XerD
COG2207	AraC-type DNA-binding domain and AraC-containing proteins
COG0438	Lipopolysaccharide 1,6-galactosyltransferase, GT1 family
COG1309	DNA-binding protein, AcrR family, includes nucleoid occlusion protein SlmA
COG0642	Signal transduction histidine kinase
COG0456	Ribosomal protein S18 acetylase RimI and related acetyltransferases
COG1609	DNA-binding transcriptional regulator, LacI/PurR family G1349—DNA-binding transcriptional regulator of sugar metabolism, DeoR/GlpR family
COG1051	ADP-ribose pyrophosphatase YjhB, NUDIX family
COG1132	ABC-type multidrug and LPS transport system, ATPase and permease component MsbA
COG0596	2-succinyl-6-hydroxy-2,4-cyclohexadiene-1-carboxylate synthase MenH/undecaprenyl monophosphate-sugar esterase UshA/YqjL
COG0583	DNA-binding transcriptional regulator, LysR family
COG0564	Pseudouridine synthase RluA, 23S rRNA- or tRNA-specific
COG1028	NAD(P)-dependent dehydrogenase, short-chain alcohol dehydrogenase family
COG1187	Pseudouridylate synthase RsuA/RluF, specific for 16S rRNA U516, 23S rRNA U2604/U2605/U2457, and tRNA(Tyr)-35
COG0834	ABC-type amino acid transport/signal transduction system, periplasmic component/domain
COG1249	Dihydrolipoamide dehydrogenase (E3) component of pyruvate/2-oxoglutarate dehydrogenase complex or glutathione oxidoreductase
COG0513	Superfamily II DNA and RNA helicase
COG1108	ABC-type Mn^2+^/Zn^2+^ transport system, permease component
COG1131	Ribosome-associated ATPase or ATPase component of an ABC-type multidrug transport system
COG0664	cAMP-binding domain of CRP or a regulatory subunit of cAMP-dependent protein kinases
COG0745	DNA-binding response regulator, OmpR family, contains REC and winged-helix (wHTH) domain
COG0561	Hydroxymethylpyrimidine pyrophosphatase and other HAD family phosphatases
COG0765	ABC-type amino acid transport system, permease component

## Data Availability

The original contributions presented in this study are included in the article. Further inquiries can be directed to the corresponding author(s).
